# Epidermal Growth Factor Receptor (EGFR)-Targeting Peptides and Their Applications in Tumor Imaging Probe Construction: Current Advances and Future Perspectives

**DOI:** 10.3390/biology14081011

**Published:** 2025-08-07

**Authors:** Lu Huang, Ying Dong, Jinhang Li, Xinyu Yang, Xiaoqiong Li, Jia Wu, Jinhua Huang, Qiaoxuan Zhang, Zemin Wan, Shuzhi Hu, Ruibing Feng, Guodong Li, Xianzhang Huang, Pengwei Zhang

**Affiliations:** 1 The Second Clinical College, Guangzhou University of Chinese Medicine, Guangzhou 510120, China; 19806841470@163.com (L.H.); dongying84523@outlook.com (Y.D.); chrisyxy1018@163.com (X.Y.); cindylee2009@126.com (X.L.); wujia_0729@163.com (J.W.); zhangqiaoxuan123@163.com (Q.Z.); 13760673961@163.com (Z.W.); 2 Guangdong Provincial Biotechnology Research Institute, Guangzhou 510663, China; ljh@gdlami.com; 3 School of Chemistry and Chemical Engineering, South China University of Technology, Guangzhou 510640, China; jhhuang2022@foxmail.com (J.H.); szhu@scut.edu.cn (S.H.); 4 Macao Centre for Research and Development in Chinese Medicine, The State Key Laboratory of Mechanism and Quality of Chinese Medicine, Institute of Chinese Medical Sciences, University of Macau, Macao SAR 999078, China; fengruibing128@126.com; 5 Guangdong-Hong Kong-Macau Joint Lab on Chinese Medicine and Immune Disease Research, Guangzhou 510120, China; 6 Guangdong Provincial Clinical Research Center for Laboratory Medicine, Guangzhou 510120, China

**Keywords:** epidermal growth factor receptor, targeting peptide, tumor imaging, imaging agents, target therapy and diagnosis

## Abstract

The epidermal growth factor receptor (EGFR) is a key target for both cancer diagnosis and therapeutic interventions. Assessing EGFR expression before therapy has become routine in clinical practice, yet current methods like biopsy and immunohistochemistry (IHC) have significant limitations, including invasiveness, limited repeatability, and lack of real-time, whole-body data. EGFR-targeted imaging has emerged as a promising alternative. EGFR-targeting peptides, owing to their favorable physicochemical properties and versatility, are increasingly being explored for a variety of applications, including molecular imaging, drug delivery, and targeted therapy. Recent advances have demonstrated the potential of EGFR-targeting peptides conjugated to imaging probes for non-invasive, real-time in vivo tumor detection, precision therapy, and surgical guidance. Here, we provide a comprehensive overview of the latest progress in EGFR-targeting peptides development, with a particular focus on their application in the development of molecular imaging agents, including fluorescence imaging, PET/CT, magnetic resonance imaging, and multimodal imaging. Furthermore, we examine the challenges and future directions concerning the development and clinical application of EGFR-targeting peptide-based imaging probes. Finally, we highlight emerging technologies such as artificial intelligence, mutation-specific peptides, and multimodal imaging platforms, which offer significant potential for advancing the diagnosis and treatment of EGFR-targeted cancers.

## Simple Summary

The epidermal growth factor receptor (EGFR) plays a crucial role in cancer and is often targeted for both diagnosis and treatment. Traditionally, EGFR levels are measured through tissue biopsies and immunohistochemistry, but these methods are invasive and cannot provide whole-body or real-time information. This review explores a promising alternative—non-invasive imaging using peptides that specifically bind to EGFRs. These small molecules are easy to produce, stable, and can be linked to imaging agents for use in imaging-based diagnostic techniques, like computed tomography (CT) and magnetic resonance (MR). Such EGFR-targeting peptides can help doctors detect tumors in real time, guide surgeries more precisely, and monitor treatment response without needing repeated biopsies. We summarize recent developments in this field, discuss the challenges that remain—such as improving stability and targeting accuracy—and highlight exciting future directions, including the use of artificial intelligence and advanced imaging technologies. This work provides important insights that may lead to safer, faster, and more accurate cancer diagnosis and treatment, benefiting both patients and the healthcare system.

## 1. Introduction

The human epidermal growth factor receptor (EGFR), a member of the ErbB family of receptor tyrosine kinases (TKs), is a transmembrane glycoprotein expressed on the surface of most human cells [1]. Upon activation by its endogenous ligands, EGFR triggers downstream signaling pathways that regulate key cellular processes, including proliferation, survival, migration, and differentiation [2]. Clinical studies have demonstrated that EGFR is frequently overexpressed or aberrantly activated in multiple cancers, making it a crucial biomarker for cancer diagnosis and a major therapeutic target [3].

Assessing EGFR expression levels prior to therapy has become a routine clinical practice [4,5]. Currently, EGFR detection primarily relies on biopsy and immunohistochemistry (IHC) [6,7]. However, these methods have inherent limitations: biopsies are invasive and unsuitable for repeated monitoring, tumor heterogeneity can lead to inaccurate results, and neither biopsy nor IHC provides real-time, whole-body EGFR expression data [8]. These limitations highlight the urgent need for an accurate, non-invasive, and real-time method for detecting EGFR expression in vivo.

EGFR-targeted imaging strategies have emerged as a promising solution. Compared to traditional biopsy and IHC, imaging-based techniques offer significant advantages, including the non-invasive, real-time, and whole-body visualization of EGFR expression. They also enable repeated monitoring without the need for additional tissue sampling and allow the assessment of intratumoral heterogeneity and distant metastatic lesions. In recent years, significant efforts have been directed towards the development of EGFR-specific imaging probes, with the goal of enhancing imaging precision, reducing background noise, and enabling the real-time visualization of EGFR-positive tumors. These probes typically comprise two key components: (1) a reporter molecule that generates signals for imaging modalities such as fluorescence, positron emission tomography–computed tomography (PET/CT), or magnetic resonance imaging (MRI), and (2) a targeting ligand that specifically binds to EGFR [9]. For example, very recently, ABY-029, an anti-EGFR affibody molecule conjugated to IRDye 800CW, was approved for clinical trial related to soft-tissue sarcomas diagnosis [10,11,12]. Currently, the EGFR-targeting molecules include tyrosine kinase inhibitors (TKIs) [13,14], antibodies [15,16,17], affibodies [10,18], aptamers [19,20], and peptides [21]. This review specifically focuses on EGFR-targeting peptides and their applications in molecular imaging. Among these, peptides offer advantages such as smaller molecular size, better biocompatibility, ease of synthesis, and improved storage stability [22,23]. Furthermore, peptides can be rapidly developed through techniques like phage display and structure-based computational design, making them an attractive alternative for EGFR-targeted imaging.

In this review, we first summarize the reported EGFR-targeting peptides and the techniques used for developing these peptides. Next, we provide an overview of the EGFR imaging techniques developed using these targeting peptides and their recent applications in tumor diagnosis and treatment. Finally, we discuss the challenges and future perspectives regarding the development and clinical application of EGFR-targeting peptide-based imaging probes. This review aims to provide guidance for the development of EGFR-targeting peptide-based imaging probes, with a particular focus on their clinical applications in fluorescence imaging, PET/CT, magnetic resonance imaging, and multimodal imaging.

## 2. EGFR-Targeting Peptides and Their Development Methods

To date, over 25 EGFR-targeting peptides have been developed using various techniques, with the majority derived from phage display. A summary of these peptides and the methods used for their development is presented in Table 1. Phage display remains the most widely used technique for targeting peptide development [24]. For example, the peptide **Pep4** (GE11, Table 1), one of the most studied EGFR-targeting peptides, was enriched from a 4 × 10^10^ pfu phage peptide library against the extracellular domain of recombinant human EGFR [25]. In addition to linear peptides, cyclic peptides targeting EGFR have also been screened using techniques like C7C phage display. For example, Furman et al. reported two novel cyclic peptides, **Pep7** and **Pep8**, for targeting EGFR and the EGFRvIII mutation [26]. Cyclic peptides offer advantages over linear peptides, including enhanced structural rigidity, improved binding affinity and selectivity, increased metabolic stability, and better membrane permeability [27].

Despite its success, display-based approaches such as phage display or ribosome display remain labor-intensive, costly, and time-consuming. With the advancement of computational algorithms and artificial intelligence, in silico approaches have emerged as powerful alternatives for designing and optimizing protein-binding peptides. Peptide sequences generated through computational methods are typically assessed for binding affinity through molecular docking and molecular dynamics simulations, which help refine peptide selection. EGFR-targeting peptides, such as **Pep12** to **Pep15**, were developed using computational methods. Recently, artificial intelligence has further enhanced the accuracy and efficiency of peptide design. For example, Chen et al. developed a computational approach that integrated deep learning with structural modeling to design highly specific β-catenin inhibitors [28].

Another promising approach in peptide design is structure-based peptide development, which leverages known protein–protein interaction motifs to create highly specific targeting peptides. By analyzing natural protein–protein interactions, researchers can identify critical binding motifs, interaction hotspots, and conformational preferences of the target protein. Then, peptide sequences that mimic key interaction sites are designed and further optimized using computational techniques. For example, **Pep18** (Table 1) was developed based on the interaction between sorting nexin and the intracellular domain of EGFR [29]. It binds to the C-terminal region of the EGFR kinase domain and effectively disrupts the interaction between sorting nexin and EGFR. This strategy allows for the rational design of peptides that mimic natural binding partners, enhancing their specificity and affinity.

Sequence optimization and modification of existing peptides represent another strategy for improving targeting efficiency. Researchers can identify key amino acid residues that influence binding affinity and specificity. Site-directed amino acid substitution can then be used to enhance peptide affinity (**Pep5** and **Pep6**, Table 1) [30,31]. Additional modifications, such as covalent cyclization, hydrocarbon stapling, and the introduction of unnatural amino acids, can also improve peptide stability and targeting specificity [32,33,34].

**Table 1:** Summary of reported EGFR-targeting peptides and their development approaches.

No.	Sequence	Targeting Selectivity	Development Approaches	Ref.
**Pep1**	HTSDQTN	EGFR WT	Phage display	[24]
**Pep** **2**	SYPIPDT	EGFR WT	Phage display	[24]
**Pep3**	QRHKPRE	EGFR WT	Phage display	[35]
**Pep4**	YHWYGYTPQNVI	EGFR WT, EGFR vIII	Phage display	[25]
**Pep5**	YHWYGYTPENVI	EGFR WT, EGFR vIII	Structure-based peptide design	[30]
**Pep6**	YHWYGYTPQNYI	EGFR WT, EGFR vIII	Structure-based peptide design	[31]
**Pep7**	CHVPGSTIC	EGFR WT, EGFR vIII	Phage display	[26]
**Pep8**	CVAAMGSTC	EGFR WT, EGFR vIII	Phage display	[26]
**Pep9**	CEKMVATHC	EGFR WT	Phage display	[36]
**Pep10**	CPSDEHHTC	EGFR WT	Phage display	[36]
**Pep11**	VLGREEWSTSYW	EGFR vIII	Phage display	[37]
**Pep12**	LARLLT	EGFR WT	Computer-aided design	[38]
**Pep13**	KYFPPLALYNPTEYFY	EGFR WT	one-bead-one-compound library	[39]
**Pep14**	STHHYYP	EGFR L858R	Structure-based in silico design	[40]
**Pep15**	HTHYYLP	EGFR L858R	Structure-based in silico design	[40]
**Pep16**	CMYIEALDKYAC (CY12)	EGFR WT	Structure-based peptide design	[41]
**Pep17**	CY12-63, CY12-81 and CY12-83	EGFR WT	Structure-based in silico design	[42]
**Pep18**	YARAAARQARAKNHVIKYLETLLYSQQQLAKYWEAFL	EGFR WT	Structure-based peptide design	[29]
**Pep19**	FMRRRHIVRKRTLRRLLQERE	EGFR WT	Structure-based peptide design	[43]
**Pep20**	AEYLR	EGFR WT	Structure-based peptide design	[44]
**Pep21**	EYINQ	EGFR WT	Structure-based peptide design	[44]
**Pep22**	PDYQQD	EGFR WT	Structure-based peptide design	[44]
**Pep23**	NYQQN	EGFR WT	Structure-based peptide design	[45]
**Pep24**	RAHEEIYHFFFAKKK	EGFR WT	Structure-based peptide design	[46]
**Pep25**	SYRRPSQIRY	EGFR WT	Antibody fragment	[47]
**Pep26**	FALGEA	EGFR vIII	Positional scanning synthetic combinatorial library	[48]
**Pep27**	CQTPYYMNTC	EGFR WT	Structure-based peptide design	[49]
**Pep28**	LLCSLYPGSSL	EGFR WT	Ribosome display selection	[50]

## 3. Construction of EGFR-Targeting Peptide-Based Imaging Probes

EGFR is a critical biomarker for cancer diagnosis and therapy. However, its clinical assessment primarily relies on invasive methods such as biopsy and immunohistochemistry (IHC), which are limited by sampling errors, tumor heterogeneity, and the inability to provide real-time information. To overcome these limitations, non-invasive molecular imaging techniques have been explored for in vivo EGFR detection.

In this part, we mainly focus on discussing EGFR-targeted imaging strategies using EGFR-targeted peptides. By integrating EGFR-targeting peptides with imaging agents, researchers have developed peptide-based probes compatible with various imaging modalities, including NIR fluorescence, MRI, and PET/CT. These probes have demonstrated strong potential as valuable tools in tumor diagnostics, therapy efficacy monitoring, surgical guidance, and therapy.

### 3.1. Fluorescence Imaging

**Figure 1:**
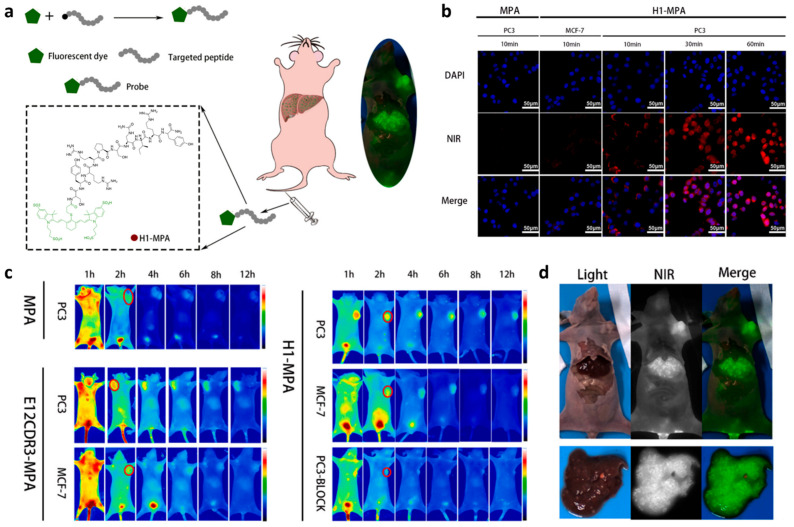
(**a**) Schematic illustration of establishing **Pep25** (H1)-based NIR probe H1-MPA for high-precision tumor imaging. (**b**) Cytofluorimetric imaging of H1-MPA in MCF-7 (EGFR negative) and PC-3 (EGFR-positive) cells. DAPI (blue) stains cell nuclei, NIR channel (red) indicates probe localization, and merged images combine both signals. (**c**) In vivo imaging of MPA, E12CDR3-MPA, and H1-MPA in subcutaneous PC-3 and MCF-7 xenograft models. The pseudo-color images display fluorescence intensity (blue: low, red: high). Red regions indicate targeted probe accumulation in tumors. (**d**) Hepatic metastasis imaging for H1-MPA. Adapted from Ref. [47] with permission from Elsevier.

**Figure 2:**
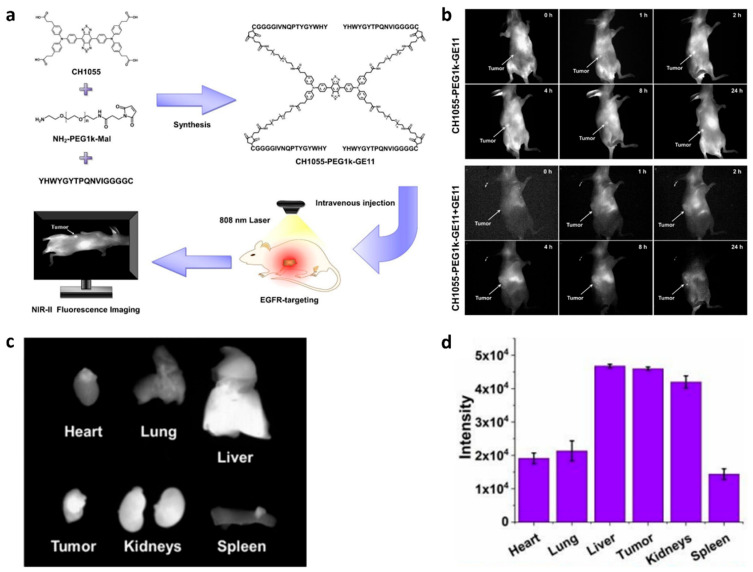
(**a**) Schematic illustration of CH1055-PEG1k-**Pep4** (GE11) design and non-invasive NIR-II fluorescence imaging of the EGFR-positive tumor in vivo. (**b**) NIR-II fluorescence imaging of H1264 tumor-bearing mice post intravenous administration of CH1055-PEG1k-**Pep4**, with or without **Pep4** competitive blockade. (**c**) NIR-II fluorescence image of the tumor and organs collected from tumor-bearing mice injected with CH1055-PEG1k-**Pep4**. (**d**) NIR-II fluorescence intensity in the tumors and major organs of tumor-bearing mice 24 h post-injecting CH1055-PEG1k-**Pep4**. Adapted from Ref. [52] with permission from the Royal Society of Chemistry.

**Figure 3:**
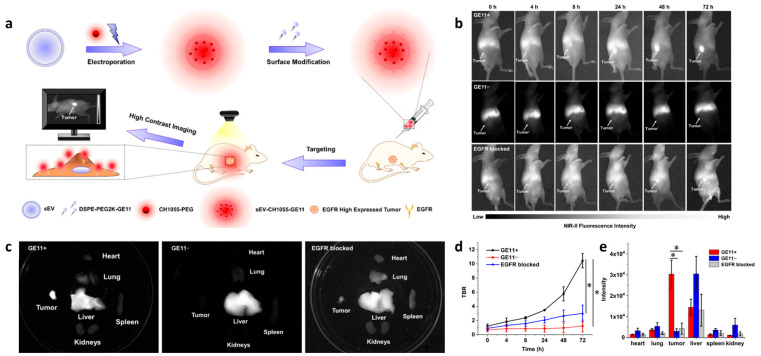
(**a**) Schematic illustration showing the establishment of the NIR-II fluorescent nanoprobe sEV-CH1055-**Pep4** for the non-invasive high-contrast imaging of the tumor with high EGFR expression. (**b**) NIR-II fluorescence imaging of sEV-CH1055-Pep4, sEV-CH1055, and cetuximab+sEV-CH1055-Pep4 (EGFR blocked group) after intravenous injection. (**c**) NIR-II fluorescence imaging of the tumors and organs obtained from the indicated mice 72 h after injection. (**d**) TBR plot of the indicated mice group during 72 h imaging, N = 3. (**e**) Fluorescence intensity of the tumors and organs collected from the indicated mice group after 72 h imaging, N = 3, *, *p* < 0.05. Adapted from Ref. [53] with permission from American Chemical Society.

Fluorescence imaging (FI) is an ideal diagnostic technique due to its high sensitivity, lack of radiation, and relatively low cost. However, a limitation of traditional fluorescence imaging is the low depth of light penetration. Near-infrared (NIR, 650–1700 nm) imaging addresses this limitation by offering low autofluorescence, deep tissue penetration, minimal light scattering, and a high signal-to-background ratio, making it an effective real-time imaging method [51]. For example, to achieve high sensitivity and high resolution imaging of EGFR-overexpressed tumors, Huang et al. developed H1-MPA, an EGFR-targeted NIR fluorescent probe using the short peptide H1 (**Pep25**), derived from the CDR3 region of an anti-EGFR nanobody [47]. It has been demonstrated that H1-MPA exhibits high EGFR affinity, low cytotoxicity, strong tumor uptake, and a high tumor-to-background ratio (TBR) (Figure 1). Additionally, in a liver metastasis model, H1-MPA was able to clearly delineate the boundary between tumor and normal liver tissue (Figure 1d). These results suggest that H1-MPA could serve as a valuable tool for EGFR-positive cancer diagnosis and monitoring tumor metastasis. In another study, Hong et al. developed CH1055-PEG1k-**Pep4**, an EGFR-targeted NIR-II probe, by conjugating the dye CH1055 with the targeting peptide **Pep4** [52]. This probe showed enhanced EGFR-targeting ability and low cytotoxicity on H1264 cells. In animal studies, it achieved a high TBR of 12.89 at 2 h, which is significantly higher than the ROSE (rapid on-site evaluation) standard (Figure 2). Additionally, the team developed a biocompatible NIR-II nanoprobe, sEV-CH1055-**Pep4**, by conjugating **Pep4** with small extracellular vesicles (sEVs) and CH1055 [53]. In vivo, sEV-CH1055-**Pep4** produced a high contrast and targeting ability in H1264 tumor-bearing mice, with a TBR of 10.43 at 72 h, which has a much longer retention time in tumor tissue than previous CH1055-PEG1k-**Pep4** (Figure 3), showing promise for the non-invasive high-contrast NIR-II fluorescence imaging of EGFR-overexpressing tumors.

The overexpression of EGFR has been implicated in the development of colorectal cancer. The NIR-based quantitative imaging tool for EGFR-positive colorectal cancer detection has been extensively studied [35,54]. Zhou et al. identified a peptide, **Pep3**, specifically capable of targeting the EGFR domain II [35]. The conjugation of this peptide with NIR fluorophore Cy5.5, a quantitative endoscope tool for EGFR detection, was developed. This study paves the way for NIR-based endoscopic imaging in EGFR-overexpressing cancer diagnosis. Subsequently, the probe was adapted into a side-viewing confocal endomicroscope, offering superior imaging speed and resolution compared to current clinical endoscopic methods [55]. Given the probe’s excellent diagnostic performance, the probe was further evaluated in a phase I clinical trial for the diagnosis of Barrett’s neoplasia [56]. Additionally, Vicente et al. synthesized three BODIPY-peptide conjugates and compared their specificity for binding to EGFR [57,58]. The results showed that BODIPY-**Pep12** conjugates bind to EGFR more effectively than other conjugates, showing it to be a promising contrast agent for the detection of colorectal cancer and other EGFR-overexpressing cancers.

**Figure 4:**
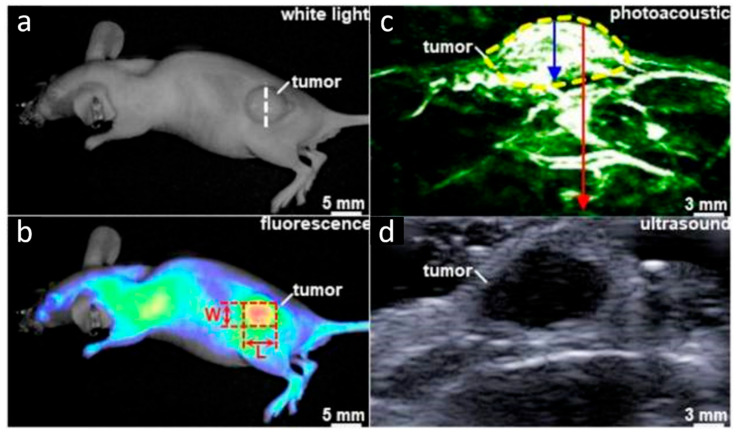
Xenograft tumor imaging using **Pep3**-KSP-IRDye800 probe. (**a**) The white light image shows the location of the human esophageal xenograft tumor in a nude mouse. (**b**) NIR FL image collected 2 h after heterodimer (**Pep3**-KSP-IRDye800) injection shows tumor dimensions (red dash) of L × W = 6.4 × 4.7 mm^2^. (**c**) Sagittal view of PA image collected along the (white dashed) line in panel (**a**) shows tumor (yellow dash) highlighted by **Pep3**-KSP- IRDye800 with 4.8 mm (blue arrow) depth and 1.2 cm (red arrow) total depth. (**d**) Ultrasound image confirming tumor structure. Adapted from Ref. [59] with permission from the Royal Society of Chemistry.

In esophageal adenocarcinoma, the expression of EGFR and HER2 is commonly elevated. Chen et al. synthesized a heterodimeric peptide containing the EGFR-targeting peptide **Pep3** and the HER2-targeting peptide KSP, and the heterodimer was further labeled with the dual-modality probe IRDye800 [59]. The probe could specifically bind to cancer cells expressing EGFR or HER2, thus achieving higher accuracy and specificity. In the xenograft animal model, this probe enabled the complementary visualization of the tumor in both planar and sagittal views (Figure 4), demonstrating promising applications in cancer-targeted diagnosis and cancer staging.

In addition to the peptide specifically binding to the extracellular domain of EGFR, Han et al. discovered that a short peptide derived from the C-terminal of EGFR (AEYLR, **Pep20**) is capable of specifically binding to the intracellular C-terminus of EGFR [44]. The team synthesized A-D-NLC, a nanostructured lipid carrier (NLC) modified with **Pep20** and NIR dye, capable of targeting EGFR-positive cells in vivo [60]. The results showed that A-D-NLC could effectively differentiate between tumor and normal tissues and demonstrated great potential for the imaging monitoring of tumor size and distribution.

Fluorescence imaging significantly enhances the contrast between tumors and surrounding normal tissues, making it a valuable tool in surgical navigation [61,62]. Li et al. developed a novel NIR fluorescent imaging probe by conjugating the peptide **Pep3** with the NIR dye Cy5.5, which was applied in the laparoscopic resection of hepatocellular carcinoma [61]. In animal studies, the probe demonstrated a higher tumor-to-background ratio compared to the control group, highlighting its potential to improve surgical outcomes by aiding in more accurate tumor detection and resection.

Notably, despite the widespread use of near-infrared (NIR) imaging in preclinical studies, its clinical translation remains limited due to the poor tissue penetration of NIR photons. As a result, NIR-based imaging is primarily restricted to the detection of superficial tumors and intraoperative guidance during surgical resection. Future developments, such as second-window NIR-II dyes [63] or hybrid imaging systems (e.g., PET/NIRF), may help address these challenges.

### 3.2. PET/CT

**Figure 5:**
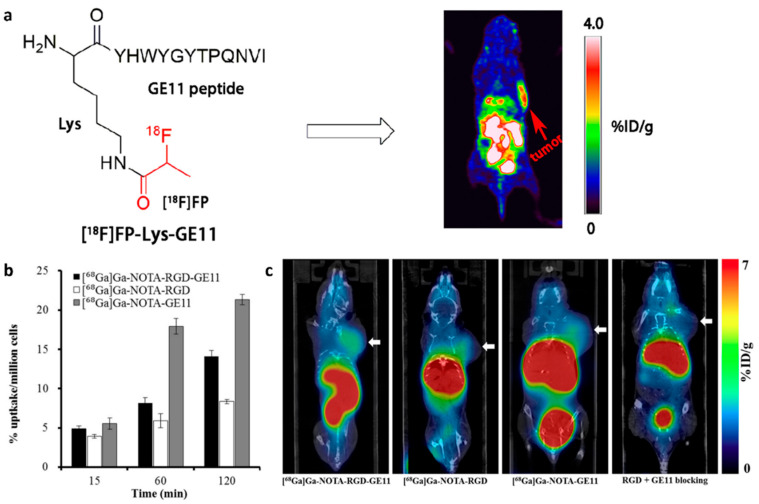
(**a**) Structure of [18F]FP-Lys-**Pep4** (GE11), a radiolabeled imaging probe for tumors with elevated EGFR expression [65]. (**b**) Cell uptake assay of ^68^Ga-NOTA-RGD-BBN, ^68^Ga-NOTA-RGD, and ^68^Ga-NOTA-**Pep4** in NCI-H292 cells. (**c**) Representative whole-body coronal PET/CT images of NCI-H292 tumor-bearing mice 2 h after the intravenous injection of 7.4 MBq of NOTA-RGD-**Pep4** (GE11) with the other three control probes. Adapted from Refs. [65,70] with permission from Elsevier.

Positron emission tomography (PET) and computed tomography (CT) are commonly used imaging techniques for tumor diagnosis and localization [64]. The incorporation of EGFR-targeting peptides into PET/CT agents offers new opportunities for the molecularly specific targeting imaging of tumors. To achieve the targeted PET imaging of EGFR-overexpressing tumors, Li et al. developed a novel PET probe by conjugating the EGFR-targeting peptide **Pep4** with 4-nitrophenyl-2-[18F]fluoropropionate ([18F]NFP) [65]. They found that [18F]FP-Lys-**Pep4** could specifically bind to EGFR-positive tumor cells and clearly delineate the tumor morphology in PET imaging (Figure 5a). Similarly, a novel SPECT agent was developed for the non-invasive imaging of EGFR-overexpressing tumors via the conjunction of ^99m^Tc with **Pep4** [66]. The new SPECT tracer also showed high EGFR specificity, favorable pharmacokinetics, and great potential for EGFR-targeted imaging. In another study, Gaenaell et al. conjugated the EGFR-targeting peptide **Pep4** to the cobalt chelator TZTPEN (N1-((triazol-4-yl)methyl)-N1,N2,N2-tris(pyridin-2-ylmethyl)ethane-1,2-diamine) to generate the SPECT probe [^57^Co]Co-TZTPEN-GE11. Although the probe exhibited excellent radiolabeling efficiency, stability, and EGFR-specific uptake in vitro, it failed to demonstrate tumor accumulation in vivo, likely due to the limited in vivo stability of the cobalt coordination complex [67]. Given that EGFR and integrin αvβ3 are often concurrently overexpressed in various malignant tumors [68,69], Chen et al. enhanced tumor imaging specificity and sensitivity by conjugating **Pep4** with the integrin-targeting peptide RGD and labeling it with ^68^Ga to create the dual-receptor PET probe ^68^Ga-NOTA-RGD-**Pep4** [70]. In an animal tumor model, ^68^Ga-NOTA-RGD-**Pep4** demonstrated higher tumor uptake, prolonged accumulation, and a greater tumor-to-muscle (T/M) ratio compared to the monomeric peptide probe (Figure 5b,c), highlighting its promising potential for dual-receptor-targeted tumor imaging using PET/CT. The dual-receptor strategy, exemplified by ^68^Ga-NOTA-RGD-**Pep4**, combines an EGFR-targeting peptide (**Pep4**) with an integrin αvβ3-binding RGD motif. This approach enhances imaging specificity by enabling concurrent binding to two highly expressed tumor receptors. It also improves sensitivity by increasing binding avidity and tumor retention, while reducing nonspecific uptake. Such synergy is particularly advantageous in tumors exhibiting the heterogeneous expression of individual targets, as dual targeting compensates for regional receptor variability, resulting in improved imaging contrast and diagnostic reliability.

### 3.3. Magnetic Resonance Imaging

**Figure 6:**
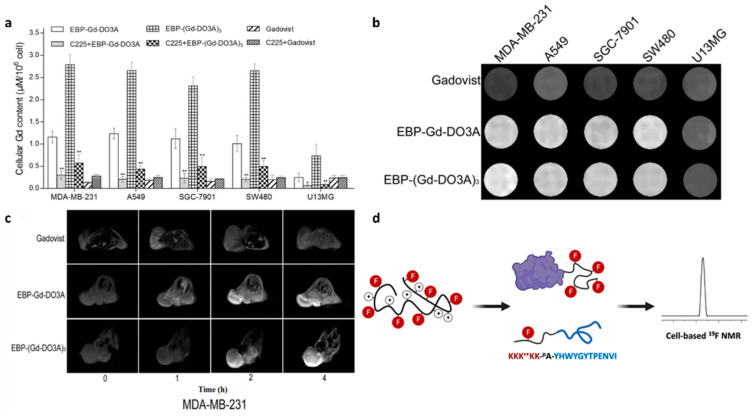
(**a**) Cellular Gd accumulation in various tumor cell lines (MDA-MB-231, A549, SGC-7901, SW480, and U13MG) after incubation with the contrast agents **Pep16**-Gd-DO3A, **Pep16**-(Gd-DO3A)_3_, or Gadovist^®^ for 12 h. *, *p* < 0.05. **, *p* < 0.01. (**b**) In vitro MRI images of various tumor cell lines (MDA-MB-231, A549, SGC-7901, SW480, and U13MG) after incubation with the contrast agents **Pep16**-Gd-DO3A, **Pep16**-(Gd-DO3A)_3_, or Gadovist^®^ for 24 h. (**c**) MRI molecular imaging of **Pep16**-Gd-DO3A and **Pep16**-(Gd-DO3A)_3_ in EGFR-overexpressing tumor cells. Adapted from Ref. [72] with permission from American Chemical Society. (**d**) Schematic illustration of the EGFR-targeting peptide **Pep5** modified with hyper-fluorination for maintaining a high signal in ^19^F NMR experiments on A431 cells. Adapted from Ref. [75] with permission from Elsevier.

Magnetic resonance imaging (MRI) is a non-invasive imaging modality that generates high-resolution, three-dimensional anatomical images [71]. Commonly used MRI contrast agents include Gadolinium (Gd), ^19^F, and iron oxide. The incorporation of EGFR-targeting peptides into these contrast agents can enhance MRI sensitivity, extend the therapeutic window, and improve diagnostic accuracy. For example, Yang et al. synthesized two novel Gd-based MRI contrast agents, EBP-Gd-DO3A and EBP-(Gd-DO3A)_3_, using the EGFR-targeting peptide **Pep16** (EBP, Table 1) [72]. These agents showed higher Gd accumulation and stronger signal enhancement in EGFR-overexpressing cells compared to the clinical agent Gadovist^®^ (Figure 6a,b), which exhibited uniform uptake across all cell types. In vivo MRI experiments demonstrated that these peptide–Gd conjugates enabled EGFR-specific tumor imaging, with enhanced signal targeting EGFR-overexpressing tumor xenografts (Figure 6c). In another study, a peptide (VGB3)-based MRI contrast agent, Gd-DTPA-VGB3, targeting VEGFR1 and VEGFR2 was synthesized and demonstrated improved breast tumor targeting and MRI performance [73]. Due to the specific binding affinity of the VGB3 peptide toward VEGFRs, Gd-DTPA-VGB3 exhibited a 2.8-fold higher contrast-to-noise ratio compared to Magnevist (355 vs. 125) and demonstrated significantly enhanced tumor accumulation in 4T1 tumor-bearing mice.

Additionally, ^19^F MRI, known for its low background signal, high contrast, and quantitative capabilities, further benefits from the use of fluorinated targeting peptides [74]. Li et al. synthesized an MRI radiotracer probe (KKK^FF^KK-βA-**Pep5**) by hyper-fluorinating the N-terminal of the EGFR-targeting peptide **Pep5**. In vitro studies showed that this probe maintained a strong ^19^F NMR signal in EGFR-overexpressing A431 cells, achieving a lower detection limit (Figure 6d) [75]. However, the in vivo performance of this probe was not evaluated in their study.

**Figure 7:**
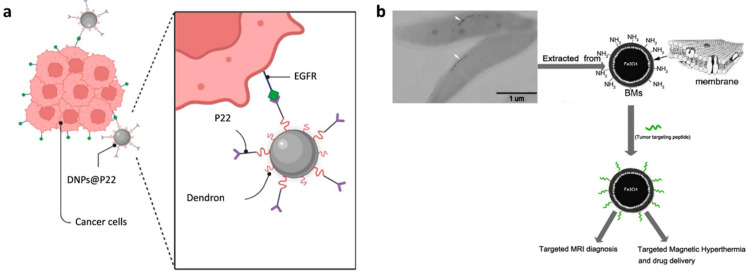
(**a**) Schematic illustration of dendronized iron oxide nanoparticles (DNPs) conjugated with **Pep5** (P22) for targeting EGFR in cancer cells. Adapted from Ref. [77] with permission from Elsevier. (**b**) Schematic representation of the synthesis process of Mag-**Pep13** NPs, a EGFR-targeted MRI diagnostic probe. Arrows indicate the magnetosome chains of magnetotactic bacteria (MSR-1). Adapted from Ref. [39] with permission from Elsevier.

Iron oxide nanoparticles (IONPs) are ideal contrast agents for enhancing MRI sensitivity and clarity due to their intrinsic superparamagnetism [76]. Conjugating targeting peptides to the surface of IONPs improves MRI sensitivity while increasing specificity and targeting accuracy for disease diagnosis. Freis et al. conjugated the EGFR-targeting peptide **Pep5** to dendrimer-based IONPs, creating MRI nanoparticles tailored for head and neck cancer imaging (Figure 7a) [77]. The results showed that the peptide-conjugated IONPs enhanced tumor cell uptake compared to non-targeted IONPs, with **Pep5** increasing internalization in EGFR-overexpressing cells. In a separate study, Xiang et al. identified a novel peptide, **Pep13**, which exhibited high affinity and specificity for both EGFR and HER2 [39]. The team functionalized the surface of magnetic nanoparticles with this peptide, synthesizing functionalized magnetosomes for the MRI of tumors (Figure 7b). They found that tumors treated with these functionalized magnetosomes exhibited significant negative contrast enhancement. Additionally, when exposed to an alternating magnetic field, these magnetosomes can be utilized for magnetic hyperthermia, further enhancing their therapeutic potential.

### 3.4. Multimodality Imaging

**Figure 8:**
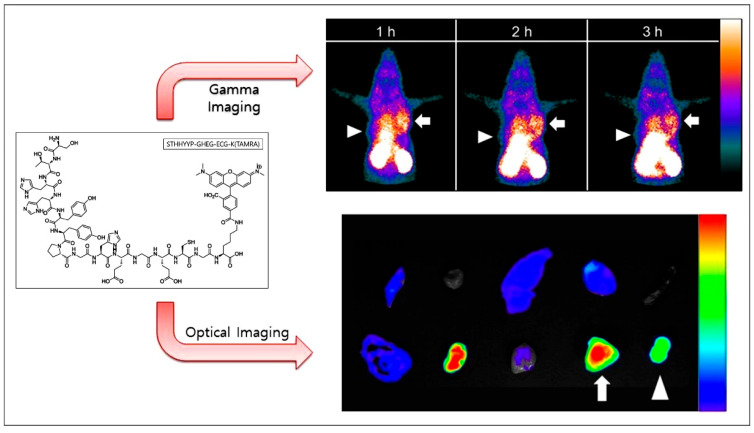
Gamma camera imaging and fluorescence optical image of **Pep14**-ECG-TAMRA in H-1975 and H-1650 tumor tissues. H-1975 tumors (arrows) exhibit significantly higher signal intensity compared to H1650 tumors (arrowheads) in both gamma (upper panel) and fluorescence (lower panel) imaging. Red denotes strong probe accumulation, while blue corresponds to background signal. Adapted from Ref. [79] with permission from Bentham science.

Multimodal imaging combines various imaging techniques to overcome the limitations of individual modalities, offering more comprehensive and accurate diagnostic information for biological systems. Kim et al. developed a novel molecular imaging probe, Tc-99m-**Pep2**-ECG-TAMRA, by conjugating the EGFR-targeting peptide **Pep2** with Tc-99m and the fluorophore TAMRA (tetramethylrhodamine) [78]. This probe enabled dual-modality imaging, integrating both fluorescence and radionuclide techniques, demonstrating the specific targeting of EGFR-positive tumor cells in an NCI-H460 xenograft model. Notably, EGFR mutations, such as the L858R mutation commonly found in non-small cell lung cancer (NSCLC), present unique challenges for cancer subtype diagnosis. Traditional methods like biopsies are invasive and prone to inaccuracies. To address this, the same team used the ^99m^Tc **Pep14**-TAMRA probe for imaging L858R-mutated EGFR tumors in the NCI-H1975 xenograft model [79]. Their findings confirmed that the probe could effectively target EGFR L858R mutations and achieve dual-modality imaging with fluorescence and radionuclides in EGFR L858R-mutated tumors (Figure 8), offering a non-invasive and dual-modality approach to monitor these mutations and improve diagnostic accuracy.

**Figure 9:**
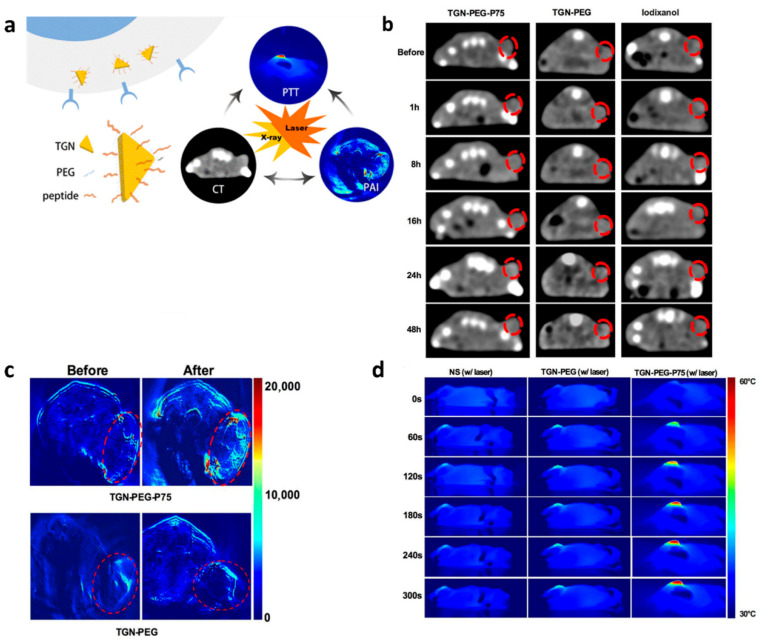
(**a**) Schematic illustration of TGN-PEG-**Pep13** (P75) for CT/PA imaging and photothermal therapy in tumor cells. (**b**) In vivo CT images of the tumor region before and at different time points post intravenous injection of TGN-PEG-**Pep13** (P75), TGN-PEG, and Iodixanol. The tumor areas are indicated by red circles. (**c**) Representative PA images of tumors in mice before and 24 h after intravenous injection with the TGN-PEG-**Pep13** (P75). (**d**) Photothermal images of HCC827 tumor-bearing mice exposed to irradiation after intravenous injection with normal saline, TGN-PEG, and TGN-PEG-**Pep13** (P75). Adapted from Ref. [80] with permission from American Chemical Society.

In a different approach, Zhao et al. developed a multimodal imaging agent using triangular gold nanoplates (TGNs), which offer strong NIR absorption and are compatible with both CT and photoacoustic (PA) imaging [80]. By conjugating the EGFR-targeting peptide **Pep13** with PEGylated TGN (Figure 9a), the TGN-PEG-**Pep13** agent exhibited superior CT imaging capabilities compared to traditional iodinated contrast agents and also provided significant photoacoustic signal enhancement. This agent’s ability to combine CT and PA imaging, along with its photothermal conversion properties (Figure 9b–d), offers a promising tool for precise tumor visualization and targeted photothermal therapy in NSCLC.

## 4. Conclusions and Future Perspectives

EGFR has become one of the most widely studied and targeted molecules in the development of cancer therapies and diagnostic strategies [2,3,6]. This review provides a comprehensive analysis of EGFR-targeting peptides and their application in various bioimaging modalities, such as fluorescence imaging, MRI, PET/CT, and multimodal imaging. These imaging techniques offer significant advantages in terms of non-invasive, high-specificity tumor detection, thereby facilitating early diagnosis, the monitoring of therapeutic efficacy, and guiding personalized treatment strategies [9].

Despite the considerable progress made, several challenges are still faced in the clinical application of EGFR-targeting peptides for tumor imaging [31,81,82]. One of the main limitations is their relatively lower binding affinity compared to antibodies and small-molecule inhibitors [31]. Nevertheless, recent developments in artificial intelligence (AI), deep learning, and structure-based peptide design offer promising avenues for overcoming this issue [28]. Through these advanced technologies, it is likely that the development of higher-affinity peptides will accelerate. In addition, peptides are prone to enzymatic degradation in vivo, which can compromise their stability, bioavailability, and imaging performance. To address these issues, anti-degradation strategies should be used to enhance proteolytic resistance and improve pharmacokinetics [83]. Furthermore, reported EGFR-targeting peptides were developed by different groups, and their amino acid sequences and structural designs varied significantly. These variations can result in distinct physicochemical properties, affecting peptide stability, EGFR binding affinity, biodistribution, and imaging performance. However, a systematic comparison of these peptides is still lacking, making it difficult to identify the most promising candidates for clinical translation. Future studies should prioritize head-to-head evaluations under standardized conditions to screen and optimize EGFR-binding peptides with superior targeting efficiency, stability, and translational potential.

In addition to the EGFR expression level, the status of EGFR mutations is critical for clinical decision-making, especially for guiding therapy with EGFR-TKIs [79]. While significant progress has been made in the development of EGFR-targeting peptides, the creation of mutation-specific peptides remains limited [79]. Future research should focus on the development of peptides that specifically target EGFR mutations, such as the 19 del, L858R, and T790M mutations commonly found in NSCLC subtypes, enabling more precise cancer diagnosis and improved therapeutic efficacy.

Furthermore, to achieve more comprehensive and accurate information about tumor characteristics, further exploration of multimodal imaging techniques is needed [78,79,80]. In parallel, theranostic probes that combine imaging and therapeutic functions, such as photothermal therapy (PTT) or photodynamic therapy (PDT), represent a promising area for future exploration [80]. The future development of imaging probes should consider integrating both diagnostic and therapeutic capabilities, achieving tumor diagnosis and treatment simultaneously.

In conclusion, this review highlights the significant potential of EGFR-targeting peptides in advancing tumor imaging and therapy. Ongoing innovations in peptide design, multimodal imaging, and theranostics will undoubtedly drive further progress in this field. By addressing the challenges related to peptide affinity, mutation specificity, and imaging resolution, future research will enhance the clinical utility of EGFR-targeting peptides, enabling more effective and personalized diagnostic and therapeutic strategies.

## Data Availability

No new data were created or analyzed in this study.
